# Impact of Syrian Conflict on the Oral Health of Adolescents: A Cross-Sectional Study

**DOI:** 10.7759/cureus.54613

**Published:** 2024-02-21

**Authors:** Wail Habal, Rana Alkattan, Mohammad Y Hajeer, Muaaz Alkhouli, Zuhair Al-Nerabieah, Tawfik Habal, Mohammed Awawdeh

**Affiliations:** 1 College of Medicine and Dentistry, Ulster University, Birmingham, GBR; 2 Clinical Dentistry (MClinDent) in Restorative and Cosmetic Dentistry, Brierley Price Prior (BPP) University, Birmingham, GBR; 3 Restorative and Prosthetic Dental Sciences, College of Dentistry, King Saud bin Abdulaziz University for Health Sciences (KSAU-HS), Riyadh, SAU; 4 King Abdullah International Medical Research Center (KAIMRC), Ministry of National Guard Health Affairs (MNGHA), Riyadh, SAU; 5 Orthodontics, Faculty of Dentistry, University of Damascus, Damascus, SYR; 6 Pediatric Dentistry, Faculty of Dentistry, University of Damascus, Damascus, SYR; 7 Orthodontics and Pediatric Dentistry, Habal Private Clinic, Paris, FRA; 8 Preventive Dental Science, College of Dentistry, King Saud Bin Abdulaziz University for Health Sciences (KSAU-HS), Riyadh, SAU

**Keywords:** well-being, oral health, dental health, syrian war, conflict, adolescents

## Abstract

Background: The Syrian conflict has had a negative impact on the psychological and overall health of adolescents. However, little is known about the oral health of those who are internally displaced.

Aims: The purpose of this study was to investigate the relationship between mental health state and self-reported oral health and habits in Syrian adolescents.

Methods: A total of 99 adolescents living in Syria were included in the study. The participants were given four questionnaires: the International Trauma Questionnaire (ITQ), the Depression Anxiety Stress Scale-21 (DASS-21), the Epworth Sleepiness Scale (ESS), and the World Health Organization (WHO) Oral Health Questionnaire for Children (2013). The relationship between self-reported oral and mental health was evaluated.

Results: Adolescents with symptoms of mental disturbances or abnormal sleep conditions were statistically more likely to self-report the health of their teeth and gums as below average, less likely to brush their teeth regularly, and reported more frequent smoking (p<0.05). Moreover, symptoms of mental disturbances and abnormal sleep conditions were statistically more likely in adolescents living in rural areas and whose parents’ education did not exceed secondary school (p<0.05).

Conclusion: Syrian adolescents reported mental disturbances, which were reflected in their poor oral health and habits. These findings confirm the need for psychiatric and oral health care programs for Syrians who remain in areas of conflict.

## Introduction

Background

Recent oral and mental health studies emphasize a biopsychosocial model, encompassing physical and mental functioning, emotional and social well-being, and self-reported symptoms as valid parameters for oral health [1]. These factors, under oral health-related quality of life (OHRQoL), serve as indicators to assess the impact of oral diseases on individuals and society, contributing to disparities in oral health and access to care. Globally, socio-economic, ethnic, and welfare-related health disparities pose a significant social problem, with factors like cost, location, availability, and safety limiting access to proper oral health care [2].

Poor oral health adversely affects oral function, self-esteem, and overall well-being, especially in children and adolescents [1]. Dental disorders like early childhood caries, anomalies, and oral pain significantly impact their perceived quality of life and overall health [3]. Using child-centered mental and oral health assessment tools reveals the interplay between psychological, oral, and dental health, providing insights beyond healthcare professionals' interpretations [4]. Acknowledging the significance of self-reported health experiences is vital for a comprehensive understanding of a child's well-being, extending beyond traditional clinical diagnoses of dental diseases.

Low OHRQoL is more common in children and adolescents from low-income and dysfunctional families, with varied impacts influenced by the environmental context and the child's adaptation. Children's reliance on parents for support significantly shapes oral health behaviors, including attitudes toward sugar consumption and hygiene routines, establishing trajectories toward good or poor oral health [5]. Dysfunctional families may struggle to provide adequate support, possess limited knowledge, and offer restricted dental care access, negatively affecting children's oral health, function, satisfaction, and self-care [6].

War significantly impacts young individuals, leading to mental trauma and depression. Research suggests psychiatric complications in children and adolescents after post-war exposure [7]. Studies on refugee children show post-traumatic stress disorder (PTSD) symptoms persist for up to five years in 53% and up to ten years in approximately 40% after war-related trauma [8-10].

The Syrian war crisis, persisting for over a decade, has led to widespread suffering, with millions internally displaced and over 80% living below the poverty line [11]. Among the internally displaced, nearly 3.8 million are children [12]. The region shows a high prevalence of PTSD among children and young adults, with a negative impact on oral health, manifested as untreated dental caries or poor oral hygiene [13]. Children diagnosed with PTSD exhibited a higher prevalence of dental caries compared to healthy children [13]. While most studies have concentrated on PTSD and depression, children exposed to conflict may also experience stress, anxiety, and sleep disorders [14]. Limited research exists on the health of Syrians remaining in conflict zones, with the majority focusing on refugees settled in other countries.

Objectives

The aim of this study was to evaluate the mental and oral health of a Syrian population of adolescents and investigate the relationship between mental health conditions and self-reported oral health and habits.

## Materials and methods

Sample selection

This was a cross-sectional study including adolescents aged 12-17 receiving treatment at the undergraduate or postgraduate dental clinics at Damascus University in Damascus, Syria. Patients were randomly selected, and parental or guardian consent was secured before enrollment. Two dentists assisted participants in completing the questionnaires. Ethical approval was granted by the College of Medicine and Dentistry in Birmingham, UK (BP0206148/250621). Exclusions comprised individuals under 12 or above 17, those medically unfit, and those unable to communicate in Arabic or English. The study included a total of 99 participants.

Tools used in the study

Four diagnostic tools were used in this study for the assessment of the mental and oral health of the participants: the International Trauma Questionnaire (ITQ) [15], the Depression Anxiety Stress Scale-21 (DASS-21) [16], the Epworth Sleepiness Scale (ESS) [17], and the World Health Organization (WHO) Oral Health Questionnaire for Children, Annex 8, 5th edition [18].

ITQ

The study utilized an Arabic version of the ITQ aligned with the 11th version of the International Classification of Diseases (ICD-11) to diagnose PTSD, disturbances in self-organization (DSO), and complex PTSD (CPTSD) symptoms. This self-reporting questionnaire consisted of 18 items and had two major subscales with three symptom clusters in each. For PTSD, the symptom clusters were re-experiencing, avoidance, and a sense of current threat. For DSO, they were affective dysregulation, negative self-concept, and disturbances in relationships. Each item is based on a four-point scale ranging from not at all to extremely applicable, and participants are asked to rate each item considering the past month. Both PTSD and DSO scores were then used in the diagnosis of CPTSD.

DASS-21

An Arabic version of the DASS-21 was used to measure three categories: depression, anxiety, and stress. This self-reporting questionnaire consisted of 21 items (seven items for each category) based on a four-point rating scale, and participants were asked to rank each item depending on its applicability over the past week, ranging from not applicable at all to applicable very much or most of the time. The depression scale assesses dysphoria, hopelessness, devaluation of life, self-deprecation, lack of interest/involvement, anhedonia, and inertia. The anxiety scale assesses autonomic arousal, skeletal muscle effects, situational anxiety, and subjective experience of anxious affect. The stress scale is sensitive to levels of chronic, nonspecific arousal. It assesses difficulty relaxing, nervous arousal, being easily upset/agitated, irritable/over-reactive, and being impatient. Scores for depression, anxiety, and stress are calculated by summing the scores for the relevant items.

ESS

An Arabic version of the ESS was employed to assess sleepiness based on observations of daytime sleep and sleepiness patterns [19]. The self-reported questionnaire consisted of eight questions related to the likelihood of participants feeling sleepy during activities requiring low effort and relative immobility. Participants rated each question on a scale of 0-3, indicating the likelihood of dozing off or falling asleep. The total score, ranging from 0 to 24, was used to measure sleepiness.

WHO Oral Health Questionnaire for Children Annex 8, 5th edition

An Arabic version of this self-reported questionnaire covered basic demographic information about the participants as well as questions about oral health and habits. The data were collected through 14 questions from the survey.

Statistical analysis

Continuous variables were presented as mean and standard deviation, while categorical variables were presented as frequency and percentage. All tests were carried out with a significance level p=0.05 and performed using SPSS Statistics for Windows, Version 25.0 (IBM Corp., Armonk, NY). Independent t-tests were used to determine the relationship between age and gender and between ITQ results. A one-way ANOVA was used to determine the relationship between age and DASS-21, ESS, oral habits, and self-reported oral health. A one-way ANOVA was also used to determine the relationship between location and parents’ education level and between DASS-21 and ESS scores. A Tukey's post-hoc comparison was conducted to identify significant differences among the groups. Chi-square tests were used to identify the relationship between gender and DASS-21, ESS, oral habits, and self-reported oral health. Chi-square tests were also used to identify the relationship between location and parents’ education level and between ITQ results, oral habits, and self-reported oral health. Chi-square tests were additionally used to identify the relationship between mental health, oral health, and habits.

## Results

Socio-demographic data

The socio-demographic data of the 99 participants, including age, gender, location, and the highest parents’ educational level, are described in Table 1. The mean age of the participants was 13.97 (±1.53) years.

**Table 1:** Socio-demographic data of the participants.

Socio-demographic data		N (%)
Gender	Male	51 (51.5%)
	Female	48 (48.5%)
Location	Urban	36 (36.3%)
	Peri-urban	26 (26.3%)
	Rural	37 (37.4%)
Parents educational level	Secondary school or less	14 (14.1%)
	High school completed	26 (26.3%)
	College/University completed	46 (46.5%)
	No adult in the household or don’t know	13 (13.1%)

ITQ findings

The ITQ questionnaire identified the prevalence of PTSD, DSO, and CPTSD symptoms among the participants as 52.5%, 51.5% and 47.5%, respectively. When participants were asked to identify the possible reason for their mental disturbance, most reported the crisis the country is going through (48.5%) as the experience with the most effect on their mental health, followed by war (37.4%), displacement (8.1%), the loss of someone close (5%) and mortar sounds (1%).

Findings on depression, anxiety and stress using DASS-21

**Figure 1:**
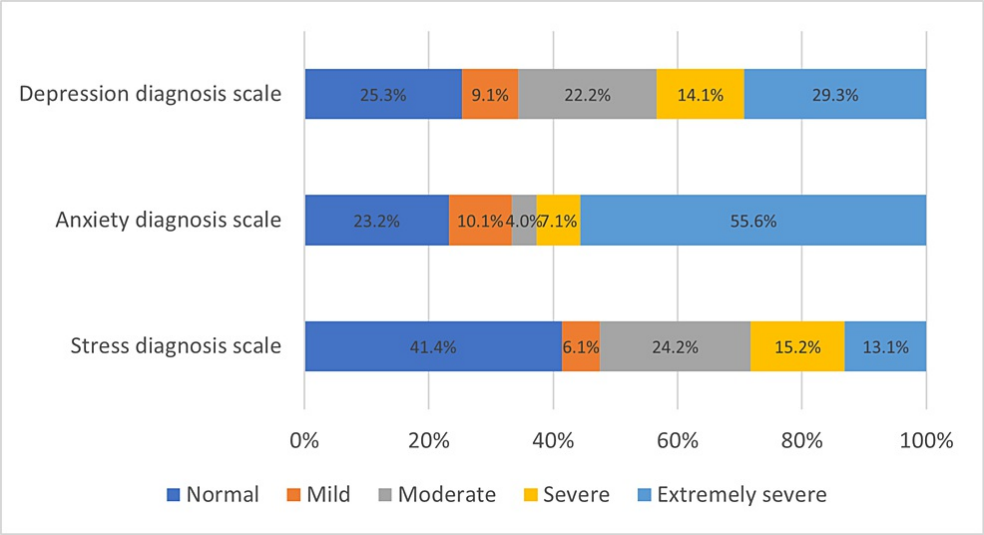
The percentage of depression, anxiety, and stress among participants.

The Depression, Anxiety, and Stress Scales are detailed in Figure 1. The majority of participants were diagnosed as having extremely severe depression and anxiety, with 29.3% and 55.6%, respectively. On the other hand, the majority of participants were diagnosed as normal on the stress scale (41.4%).

Findings on sleep quality using Epworth Sleepiness Scale

The findings on sleep quality among the participants demonstrated that 57.6% had normal sleep quality, 14.1% had some disturbances in sleeping, and 28.3% had abnormal sleep problems.

General findings on oral hygiene

**Figure 2:**
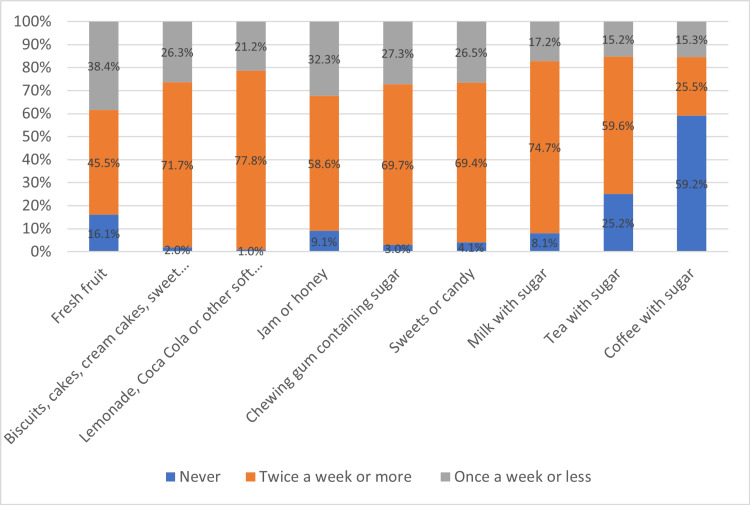
The frequency of consumption of sugar-containing foods and drinks among participants.

Most participants (49.5%) reported daily teeth brushing, and 87.9% used a toothbrush. Dental floss, chewsticks, and wooden toothpicks were used by 4%, 3%, and 1%, respectively. Regarding dentist visits, 64.6% went in the last year, primarily for issues (53%) or routine care (47%). Toothache or discomfort was reported by 79.8% in the past 12 months. Figure 2 illustrates the self-reported condition of participants' teeth and gums.

Diet and smoking

Most participants consumed sugar-containing items like sweets, gum, candy, and sugary milk multiple times weekly (Figure 2). Smoking was infrequent, with 79.8% reporting never smoking, 10.1% occasional smoking, and 10.1% smoking several times weekly or more.

Influence of socio-demographic data on mental health and habits

Age

No significant relationship was found between participants’ age and mental health disturbances, oral habits, or self-reported oral health. However, smoking among participants aged 17 years was statistically more significant (p<0.05).

Gender

No significant relationship was found between participants’ gender and mental health status or self-reported oral health. However, consumption of chewing gum with sugar, lemonade, Coca-Cola, and other soft drinks and smoking were statistically higher in males than females (p<0.05).

Location

Results of the one-way ANOVA demonstrated that participants living in rural areas demonstrated statistically greater depression, anxiety, stress, and sleep disturbance compared to those living in urban or peri-urban areas (p<0.05, Table 2). Participants living in rural areas also demonstrated statistically greater PTSD, DSO, and CPTSD compared to those living in urban and peri-urban areas (chi-square = 11.92, 10.55, and 13.35, respectively, p<0.05) and described the health of their teeth and gums as poor or less (chi-square = 21.98 and 28.22, respectively, p<0.05). Furthermore, they were statistically less likely to use a toothbrush (chi-square = 10.15, p<0.05).

**Table 2:** The relationship between participants’ location and parents’ education level and between DASS-21 and ESS scores. ^a,b,c^Different lowercase letters indicate significant differences within rows for both location and parents’ education level. ***P-value < 0.001. DASS-2: Depression Anxiety Stress Scale-21; ESS: Epworth Sleepiness Scale.

Mental health	Location						F	p-value	Parents’ education level								F	p-value
	Urban		Peri-urban		Rural				No adult in household		Secondary school/less		High school		College/University			
	M	SD	M	SD	M	SD			M	SD	M	SD	M	SD	M	SD		
Depression scale (0-21)	6.3^a^	5.18	9.2^a^	5.18	13.3^b^	5.77	15.73	***	10.5^a,b^	5.16	14.7^a^	5.62	10.8^a,b^	5.30	7.3^b^	6.03	6.87	***
Anxiety scale (0-21)	5.9^a^	5.09	9.3^b^	4.83	12.4^c^	5.70	13.51	***	10.5^a,b^	4.18	14.2^a^	5.92	10.1^a,b^	4.59	6.8^b^	5.88	7.61	***
Stress scale (0-21)	6.2^a^	4.99	8.9^a^	4.55	12.4^b^	5.60	13.25	***	10.6^a,b^	3.75	13.9^a^	5.78	10.0^a,b^	5.17	7.0^b^	5.51	6.96	***
DASS-21 score (0-63)	18.4^a^	14.88	27.4^b^	14.10	38.1^c^	16.61	14.95	***	31.6^a,b^	12.32	42.8^a^	16.72	30.9^a,b^	14.69	21.1^b^	17.10	7.51	***
Mean ESS score (0-24)	5.1^a^	4.92	7.7^a^	4.78	12.0^b^	5.49	16.92	***	8.5^b^	3.93	14.4^a^	4.22	8.9^b^	4.49	6.1^b^	6.19	8.86	***

Parents’ Education Level

Results of the one-way ANOVA demonstrated that participants with parents having reached secondary school education or less had statistically greater depression, anxiety, stress, and sleep disturbances (p<0.05, Table 2). Participants with parents having secondary school education or less also demonstrated statistically greater PTSD, DSO, and CPTSD compared to those whose parents completed college or university (p<0.05). Participants who had at least one parent with a college or university degree were statistically more likely to describe the health of their teeth and gums as good or above (chi-square = 24.80 and 14.33, respectively, p<0.05) and were statistically more likely to use a toothbrush (chi-square = 22.12, p<0.05). Furthermore, they were statistically more likely to have never smoked (chi-square = 42.37, p<0.05).

Influence of mental health on oral habits

Participants diagnosed with extremely severe depression, anxiety, or stress or those with PTSD, DSO, CPTSD, or abnormal sleep disturbances self-reported the health of their teeth and gums as average, poor, or less and were less likely to brush their teeth (p<0.05). Conversely, participants diagnosed as having normal mental health were statistically more likely to describe their teeth and gums as good or above (p<0.05). Moreover, participants diagnosed with extremely severe depression, anxiety, or stress and those with abnormal sleep disturbances were statistically more likely to report smoking several times a week or more (p<0.05).

## Discussion

The Syrian war, one of the most tragic humanitarian crises of this century, has led to millions of Syrians being internally displaced within their own country. The conflict has particularly impacted towns and villages, prompting residents to seek refuge in larger cities like Damascus. This movement has created a diverse sample of children and adolescents affected by the war. The destruction of the healthcare system and social structures and the economic crisis resulting from the conflict have left many without access to proper medical and dental assistance.

The results of the ITQ demonstrated that nearly half of the participants had symptoms of PTSD, DSO, and CPTSD. This is in accordance with many studies looking at Syrian children and adolescents who were either internally displaced or settled in other countries, with the prevalence of PTSD reported to be as high as 45.6% [20]. Adjustment to a new environment can be stressful, especially when the child has already suffered the loss of a childhood home, neighborhood, family, and friends. The comorbidity of PTSD with other mental disturbances has been previously reported [14], but very few have explored the prevalence of CPTSD. CPTSD was introduced in the 11th edition of the International Classification of Disorders and Related Health Problems and is meant to describe a more severe array of symptoms, which include not only PTSD but also the adverse effects that trauma can have on self-organization, particularly when the traumatic experience is of a prolonged or repeated nature. The high occurrence of CPTSD in the included sample is worrying since these individuals likely exhibit not only the signs and symptoms of traditional PTSD but also problems such as heightened emotional responses, feelings of worthlessness, and persistent difficulties in sustaining relationships or feeling close to others. Generally, the prevalence of psychological conditions is greater in studies from conflict areas, which is a key distinctive feature between PTSD and CPTSD, as the latter is caused by trauma that the individual perceives as being difficult to escape or life-threatening, both of which are strong predictors of psychopathology [21].

Moreover, the results of the DASS-21 demonstrated that most participants showed symptoms of depression and anxiety but not stress. Unlike ITQ results linked to traumatic events, depression often arises from prolonged adverse living conditions, while anxiety stems from anticipating future threats [22]. Notably, 48.5% attributed their mental distress to the country's crisis and 37.4% to war. Depression is characterized by a lack of positivity and energy, while anxiety involves distress and difficulty relaxing, commonly associated with tragic events and highly prevalent among refugees. Syrian refugee depression rates surged to 44% from 6.5% pre-war [23]. Stress perceived more from family separation and seeking refuge abroad was less pronounced in participants staying with families in their home country, benefiting from protective factors like familiar routines and family networks [24]. Consequently, the majority were classified as normal on the stress scale, emphasizing the role of daily stressors in shaping resilience to psychological distress among internally displaced versus externally displaced war-affected children.

In assessing the impact of sociodemographic factors on war-affected children and adolescents, age and gender did not exhibit a significant influence on key mental health outcomes. Current findings lack consensus, with no widespread agreement on specific age or gender groups being at greater risk [25]. Conversely, location and parental education emerged as robust indicators of poor mental health. The expected correlation is attributed to the heightened exposure to war-related sights and sounds in rural areas compared to urban or peri-urban settings, known to predict mental health issues [26]. In contrast to a meta-analysis reporting lower prevalence rates among child and adolescent refugees (a 22.7% prevalence of PTSD, 13.8% of depression, and 15.8% of anxiety disorders [27]), the present study indicates higher rates, likely due to the subjects remaining in the conflict-ridden country without seeking refuge elsewhere. Internally displaced populations, as revealed by a systematic review of 38 studies, demonstrate substantially higher prevalence rates for PTSD, depression, and anxiety at 88%, 80%, and 81%, respectively [28]. Additionally, children and adolescents with parents of lower education levels experience worse mental health [29]. Parents with higher education likely possess increased awareness of potential mental disturbances in their children, understand available systems and intervention needs, and consequently have better access to resources, providing more stability for their children.

The well-established connection between the psychological effects of war on children and adolescents and their oral health and social functioning is evident. Exposure to trauma and violence during wartime correlates with posttraumatic stress, poor sleep quality, and diminished perceived health status [30]. In this study, participants with poor mental health, residing in rural areas, and having parents with lower education levels were more likely to perceive their teeth and gum health as subpar. They were statistically less inclined to seek dental care or practice regular toothbrush use. These health disparities are partly attributed to limited access to dental care and the negative impact of mental health on oral hygiene motivation. Urban settings typically offer advantages over rural areas, encompassing better health resources, educational services, and infrastructure. Additional factors such as a scarcity of dental professionals, insufficient facilities in war-torn areas, high costs, and transportation difficulties may also contribute to these findings. Moreover, parents with higher education likely possess superior knowledge, fostering favorable attitudes and better oral health practices [31].

In this study, statistically significant sleep disturbances were reported by children and adolescents from rural areas and those with less educated parents. Additionally, older male adolescents from families with poor parental education showed a statistically higher prevalence of smoking. Participants diagnosed with extremely severe depression, anxiety, or stress, along with abnormal sleep disturbances, also exhibited a statistically greater frequency of smoking. This aligns with prior research indicating that smoking is a prevalent behavior among children and adolescents in war-affected areas, either as a response to environmental conditions or as a coping mechanism [29]. Adolescents experiencing poor mental health often display disrupted sleep, fear, aggression, and behavioral issues. Moreover, internally displaced children and adolescents generally exhibit these problems more than the local population [32]. Furthermore, Syrian boys, particularly those who have witnessed traumatic events and experienced conflict, tend to use smoking as a means to regulate emotional and mental imbalances [33].

This study is constrained by a small participant pool due to limitations in manpower and time for conducting research in Syria. Challenges in patient recruitment and their willingness to participate further contribute to this limitation. Moreover, the focus on participants aged 12 to 17 excludes those outside this age range. Findings also rely on self-reported measures, lacking input from parental and medical expert perspectives. Consequently, caution is advised in interpreting the results due to restricted generalizability.

## Conclusions

The study's findings highlighted the urgent need for psychiatric and health support during and after war-related exposures, as well as for long-term mental health care for young people living in war-torn countries. Self-reported outcome measures can assess mental illness and oral health, reducing the risk of bias, as found by the study. Additionally, the study found that considering and reporting the views of the participating children and adolescents increases public accountability. The study's authors conclude that a more profound understanding of the everyday difficulties of internally displaced children and the impact of war on their psychosocial and overall oral health is essential for policymakers to improve the wellbeing of children and adolescents.
